# An Integrative Model of In-Hospital and Out-of-Hospital Nursing Care for Non-Suicidal Self-Injury: A Narrative Review

**DOI:** 10.3390/brainsci13030466

**Published:** 2023-03-09

**Authors:** Ruili Zhang, Jianbo Lai, Huafen Wang

**Affiliations:** 1 Department of Psychiatry, First Affiliated Hospital, Zhejiang University School of Medicine, Hangzhou 310003, China; 3090103927@zju.edu.cn; 2 Department of Nursing, First Affiliated Hospital, Zhejiang University School of Medicine, Hangzhou 310003, China; 2185015@zju.edu.cn

**Keywords:** non-suicidal self-injury, adolescent, epidemiology, risk factor, nursing care

## Abstract

Background: Non-suicidal self-injury (NSSI) refers to the intentional and repeated physical trauma of an individual without explicit suicidal intent, which has negative effects on the physical and mental well-being of an individual, especially for adolescents. Timely and accessible nursing care may play an important role in the survival and rehabilitation process of NSSI. Methods: In this review, we systematically discuss the nursing care of NSSI behavior and provide recommendations based on an integrated nursing model for NSSI management. Results: As reported in previous studies, a variety of factors can contribute to NSSI behavior, such as personality traits, current psychological status, history of mental illnesses, as well as family and social factors. In-hospital care is the most necessary and effective care during acute episodes of NSSI behavior. To effectively manage inpatients with NSSI behaviors, nurses should first understand the motivation of NSSI, and provide comprehensive and multi-level care through nurse-patient communication, individualized psychological care, and multidisciplinary cooperation with other professionals. While the purpose of out-of-hospital nursing is to reduce the frequency of NSSI behaviors by screening high-risk individuals, providing psychological support, promoting health education, and improving self-awareness. Conclusions: An integrative model of in-hospital and out-of-hospital nursing care can help improve the clinical management and long-term prognosis of patients with NSSI and minimize the risk of suicidal ideation or suicidal behavior.

## 1. Introduction

### 1.1. The Concept of NSSI

Non-suicidal self-injury (NSSI) and suicide are both intentional acts of self-destruction but are different. The current concept of NSSI refers to the behavior of individuals who intentionally and repeatedly inflict non-fatal trauma to their bodies without explicit suicidal intent [1]. Common manifestations of NSSI include skin cutting, carving, biting, scratching, hitting the head, and even interfering with wound healing [2,3]. However, there is a sex-specific difference in NSSI patterns. That is, men are more likely to hit or burn themselves, while women are more prone to cutting and scratching the skin [2]. Overall, NSSI behaviors are more commonly seen in adolescents.

However, it was not until the publication of the Diagnostic and Statistical Manual of Mental Disorders, Fifth Edition (*DSM-5*) in 2013 that NSSI was formally defined as a type of mental disorder [1]. At the very beginning, little attention has been paid to NSSI behavior when its conception was first initiated. The diagnosis of NSSI did not appear in any official diagnostic manual as a separate mental disorder, and it was not even defined as a symptom affiliated with mood disorders or neurosis [4]. In contrast, NSSI behavior was once regarded as a transient out of emotional control or just a common manifestation of “adolescence rebellion”. In earlier studies, self-harming behavior was even considered one clinical feature of organic disorders such as intellectual disability, encephalitis, and acute poisoning [5]. In extant literature, researchers have often differentially nominated this behavior as “intentional non-lethal self-injury” or simply “self-injury”. In this review, we will uniformly use the term NSSI. According to *DSM-5*, the diagnostic criteria of NSSI require that a patient has engaged in self-injury without suicidal ideation for at least 5 days in the past year; which could not be explained by psychosis, delirium, or substance use/withdrawal [1]. Particularly, NSSI is always preoccupied with the individual and driven by negative feelings.

Despite the high prevalence of NSSI worldwide, public attitudes toward NSSI vary in different counties. There are fewer NSSI-oriented studies in Eastern countries (e.g., China) than in Western countries [6,7]. This phenomenon was partially explained by the stigma of mental illness in the traditional culture of the East [8] and the negative attitude of Confucianism to negative emotions [9]. Nonetheless, exploring effective nursing care for patients with NSSI behaviors may help to promote the recovery of NSSI and reduce the suicidal risk.

Adolescence is a critical developmental stage in lifespans. On the one hand, adolescents need to transition from being dependent on their parents to being relatively independent [10]. This process is accompanied by many unpredictable difficulties and choices in academic and interpersonal relationships. On the other hand, a series of physical and psychological changes will occur during adolescence, and these changes may disrupt their physical and emotional balance, thereby predisposing them to a higher risk of emotional and behavioral abnormalities [10]. Although most adolescents are capable of self-regulation and seeking help from others, some are at risk of developing NSSI behaviors due to a variety of internal and external factors (explained in detail below). If not timely intervened, some individuals with NSSI behavior may suffer from a higher risk of suicidal ideation and behavior [11].

In this review, we first summarized the epidemiological picture of NSSI, its relationship with suicide, and potential risk factors (e.g., alexithymic personality traits, history of mental disorders, family and social determinants) [12,13]. Based on previous findings, we proposed an integrative model of in-hospital and out-of-hospital nursing care for patients with NSSI. Most importantly, our review aims to emphasize the essential role of nurses in NSSI management.

### 1.2. Epidemiological Picture of NSSI

It is estimated that approximately 10% of the world’s population has experienced at least one NSSI behavior [14], most of which happens in adolescence. More than 20% of adolescents have NSSI behaviors, which are explained by self-punishment, relieving negative feelings, or even expressing their unhappiness to others [4]. Epidemiological studies have shown that the lifetime prevalence of at least one NSSI behavior among adolescents was about 17–18% in a community sample, of which the proportion meeting diagnostic criteria was about 1.5–6.7%. In a sample of adolescents with any mental illnesses, the lifetime prevalence of at least one NSSI behavior was as high as 60%, and the recurrence rate of NSSI behavior was about 50% [15]. According to previous studies, 5.1–24.0% of patients with NSSI reported self-injurious behavior before the age of 13 [16,17]. Longitudinal retrospective studies revealed that the incidence of NSSI behavior decreased gradually from 15 to 29 years of age [18], with the peak in the age range of 15–16 years old, and it showed a downward trend at 18 years old and afterwards [19]. In addition, compared to those without NSSI, adolescents with a history of NSSI were more likely to suffer from other psychiatric disorders currently or subsequently, such as substance abuse [20] and eating disorders [21].

Indeed, it is noteworthy that NSSI behaviors can occur in non-psychiatric patients or be co-morbid with psychiatric disorders, such as mood disorders, substance abuse, generalized anxiety disorder, and post-traumatic stress disorder [22,23,24]. Although NSSI behaviors have been once reported to be closely associated with early-onset psychiatric disorders such as schizophrenia and autism spectrum disorder [25,26], there are few studies on NSSI behaviors in children. Knowledge of NSSI behaviors in children can be obtained in a comprehensive review [27]. There are only a few studies on NSSI in children from China, most of which focused on the association between childhood abuse and NSSI behaviors in subsequent developmental stages [28,29]. For adults, most of the research in China was carried out among college students. Around the world, relevant studies involved a wider age range. A retrospective study of adults aged 19–92 years old found that 5.9% had experienced NSSI behavior (but not necessarily in adulthood) [30]. In a German study, a history of NSSI was reported in 3.1% of the 2509 adults in the study, 0.3% of which met the *DSM-5* diagnostic criteria [31].

### 1.3. The Relationship between NSSI and Suicide

NSSI and suicide (including suicidal ideation and suicidal behavior) are intrinsically different, but they often occur simultaneously or sequentially [32]. Therefore, it is necessary to distinguish suicidal ideation/behavior from NSSI, because their consequences are not the same, and there may be a strong causal correlation between them. That is, NSSI can not only cause lasting bodily harm but also predict suicidal behaviors in later life [33]. Indeed, studies have shown that adolescents with NSSI behaviors are more likely to develop suicidal ideation and behavior than their peers who have no NSSI behaviors [11]. More intuitively, 33–70% of adolescents with NSSI behaviors had at least once experienced suicidal ideation [34,35]. According to another study, about 10.9% of adolescents with NSSI behaviors had at least one suicide attempt within one year of follow-up after receiving regular medical treatment [36].

These data suggest that although NSSI itself does not equate to suicide, it can become the determinant basis for the further development of suicidal ideation and suicidal behavior in patients [22,37]. In addition, Nock and his colleagues also found that a long history of NSSI was predictive of an individual’s suicidal behavior [35]. The more severe NSSI behaviors, reflected by a higher frequency or a more variety in patterns, were indicative of the higher risk of suicide; in other words, NSSI behavior can be regarded as a “practice” of suicide [2,32].

Given the specific relationship between NSSI and suicide, and both involve intentional self-injury, some researchers have proposed the concept of a continuum, that is, uninterrupted NSSI behavior can lead to suicide [38]. Moreover, other researchers believe that the third variable such as a confirmed diagnosis of mental disorders [39], higher levels of psychological stress [38], and dysfunction of the neurotransmitter system [40] can mediate the transition from NSSI behavior to suicide. However, the above theories remain as hypotheses and have not been further verified. It is noteworthy that the independent predictive efficacy of NSSI for suicide was more suitable in individuals with NSSI accompanied by major depression, feelings of hopelessness, and inferiority, according to several comprehensive models in earlier years, which also proposed that NSSI and suicide might share overlapping risk factors (See below) [41].

## 2. Risk Factors of NSSI

**Figure 1:**
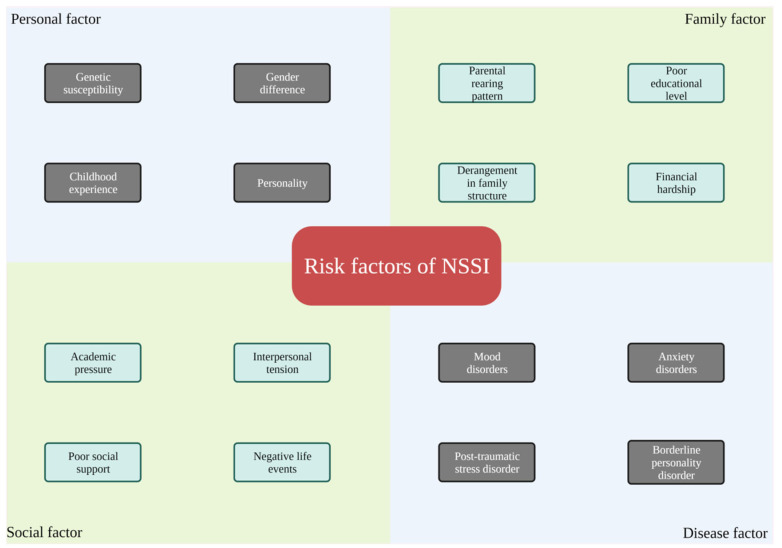
Risk factors that may contribute to NSSI behavior. In this review, we identify four groups of risk factors including personal, family, social, and disease determinants.

Risk factors of NSSI are multifaceted, including individual-specific and external factors (Figure 1). Individual factors such as genetic susceptibility, sexual difference, and personality characteristics are internal determinants that are stable and difficult to change (at least in a short period); while external factors can be intervened with via various approaches. Notably, the factors mentioned above, as well as their synergistic effects, are not necessarily determining factors for NSSI behavior. To clarify the specific cause of NSSI behavior in patients, we need to identify different factors from both clinical and non-clinical aspects, with full consideration of the patient’s childhood experience and personal characteristics.

### 2.1. Individual Factors

#### 2.1.1. Gender Difference

The incidence of NSSI behavior in adolescents varies by gender, and some studies indicate that teenage girls aged 16–19 have a relatively higher probability of NSSI behavior [42]. However, based on a comprehensive analysis of the available research findings, gender is not the determinant factor affecting the incidence of NSSI behavior in adolescents, because the epidemiological findings obtained in different regions are not consistent. For example, a study conducted in China showed that the incidence of NSSI in men was greater than that in women [43]; another study conducted in Iran reported similar findings [44]. However, other studies have shown that there is no difference in the prevalence of NSSI between men and women, but women with NSSI behaviors tend to be associated with a higher risk of suicide [45]. In addition, sexual minorities are more likely to develop NSSI [46,47,48], which may be related to less family and social support for this group [49]. However, there are still few studies on this population, and no conclusion can be drawn from the available evidence.

#### 2.1.2. Personality Characteristics

When absent from mental disorders, personality factors may be an important cause of NSSI behavior in adolescents. Studies have shown that adolescents with specific personality psychological characteristics such as difficulty in emotional regulation have a higher probability of NSSI behavior [50]. Emotion regulation is the internal and external processes responsible for monitoring, evaluating, and changing emotional responses, which are time-intensive and related to the individual’s will to achieve goals [51]. Dysregulation of emotions may lead to biased evaluations of emotional stimuli and further affect the proper process of emotion regulation, thereby making it impossible for individuals to adjust their emotions in time and coordinate their feelings with the environment [51]. Disruption in emotional regulation itself will increase the probability of NSSI behavior in adolescents; on this basis, if the individual has experienced a higher level of subjective pain experience and shows poor tolerance to pain, the probability of NSSI will also increase [52,53]. In addition, personality traits including introversion, inferiority complex, impulsiveness, and irritability are related to the occurrence of NSSI behaviors [20].

### 2.2. History of Mental Disorders

The history of mental disorders is an important factor in the occurrence of NSSI. Among various types of mental disorders, mood disorders (especially depressive disorder) are an independent risk factor for NSSI. Other mental disorders that may lead to NSSI include generalized anxiety disorder, phobia disorder, post-traumatic stress disorder, and borderline personality disorder [3,6,23]. A survey conducted in China showed that about 50% of patients diagnosed with depressive and bipolar disorders had NSSI behaviors in the past year [3]. According to a recent study, depressive disorders and anxiety disorders are the two main mental disorders that lead to NSSI behavior in Chinese adolescents [54]. It should be noted that a diagnosis of borderline personality disorder or corresponding clinical symptoms in patients with NSSI is correlated with an increased risk of suicidal ideation or behavior, which may be associated with higher levels of impulsivity and hopelessness [11,32,55]. A relevant research in China also further suggested the mediating role of personality disorders in the occurrence of NSSI behavior in individuals [56]. Therefore, when providing care, nurses should pay special attention to patients with personality disorders.

### 2.3. Family Factors

#### 2.3.1. Familial Inheritance

Not much is known regarding the genetic basis of NSSI behaviors in adolescents. Familial inheritance is an important cause of NSSI behavior in individuals, especially for adolescents. Notably, self-injurious behaviors in individuals with genetic intellectual disability syndromes may be different in nature compared to NSSI behaviors in patients with mood disorders. Children of patients with depressive disorders are more likely to exhibit NSSI behaviors [57]. A possible explanation is that adolescents mostly tend to learn the emotion regulation patterns and behaviors from their parents, in addition to genetic vulnerabilities.

Several studies have indicated the associations between the variations in glutamate and serotonin transporters and NSSI behaviors in adolescents [58,59]. Although these results need verification, it suggests the polygenetic underpinnings may play roles in the occurrence of NSSI.

#### 2.3.2. Other Family-Related Factors

To some degree, NSSI behavior in adolescents is one of the manifestations of their desire for seeking attention from other family members. In other words, the purpose of adolescents’ NSSI behaviors may not be self-injury itself, but to gain external attention through this extreme way to satisfy psychological needs [50].

Studies have shown that adolescents’ family education patterns are closely related to the occurrence of NSSI behaviors. This is because adolescents are in a critical period of character development, their ability to control emotion and behaviors is relatively weaker than adults, and their attention and mood are easily influenced by changes in the external environment. Therefore, if the family education model cannot meet the psychological characteristics of adolescents in this special period, it may cause a rebellious mentality and manifest as transient depression, irritability, or aggressive behavior [49]. A previous systematic review has shown that childhood abuse was positively associated with the occurrence of NSSI, and women who experienced childhood abuse were more likely to develop NSSI than men [60]. A questionnaire research on Chinese middle school students has also shown that childhood abuse was associated with NSSI behaviors during adolescence [28,29,61]. In addition, parents’ over-indulgence or emotional neglect can also lead to maladaptation of adolescents to the environment, resulting in an inferiority complex, which, in turn, induces NSSI behaviors [46].

The disorganization of the family structure is also an important reason for NSSI behavior in adolescents. Family is an important source for adolescents to obtain emotional support, and a well-organized family is also a necessary condition to form a healthy personality. Compared with adolescents with a complete family structure, children from single-parent families are more likely to have negative emotions, such as depression and anxiety, and tend to have rebellious thoughts and behaviors, which are all related to the occurrence of NSSI behaviors [54,62].

In addition, other studies have shown that adolescents born and raised in families with lower educational and economic levels have a higher probability of developing NSSI [62]. This may be because such families cannot provide in-time psychological or financial support to adolescents [49], resulting in adolescents not being able to adapt to the environment well, suffering from unhealthy emotions for a long period, and finally relying on NSSI behaviors as a way for emotional catharsis.

### 2.4. Social Factors

Social factors that affect the incidence of NSSI in adolescents mainly include the social environment and negative life events. The social environment includes the realistic environment and the online Internet environment.

School is the main living environment of teenagers away from family, and the cognitive development and behavioral habits of teenagers are greatly affected by the school environment. Studies have shown that the contributing factors of NSSI behavior in adolescents include excessive academic pressure, exposure to school bullying and cyberbullying, and poor interpersonal relationships [54,63,64]. Notably, NSSI behaviors in adolescents can also affect their peers and “spread” on the campus. The influence of the Internet environment on the cognitive and behavioral patterns of adolescents is subtle. Some online articles or e-books have detailed descriptions and renderings of NSSI and its similar behaviors. For adolescents who lack self-control ability, it is possible to imitate this behavior in pursuit of “different” or “fashion”. If adolescents are absent from correct guidance at this stage, NSSI behaviors may be maintained for a long time and may eventually become part of a mental disorder [65].

Negative life events encountered by adolescents, such as natural disasters and other severe traumatic events, are also an important cause of NSSI behaviors [66]. On the one hand, these events can directly cause depression, anxiety, and other negative emotions closely related to NSSI, and may induce aggressive behavior in adolescents [67]; on the other hand, these negative events may lead to post-traumatic stress disorder in those who are fragile and sensitive, which is also closely related to the occurrence of NSSI behaviors [23]. In addition, the lack of social support in adolescents after traumatic events is also a contributing factor to NSSI behaviors. Studies have shown the mediating effect of lack of social support in the association between childhood abuse and NSSI behavior in adolescence [61].

## 3. Nursing Care for NSSI

The high incidence, early-onset age, and adverse outcomes of NSSI have become a significant challenge to the national healthcare system. Given the various factors associated with mental health, nursing care for patients with NSSI should be an integration of a multi-disciplined, multi-faceted, and multi-level issue. In-hospital nursing care for NSSI focuses on the immediate and effective management of NSSI behaviors that may occur in the inpatient ward or develop into suicidal ideation and even suicidal behavior, while out-of-hospital care is a relatively broader concept. The short-term outcome and long-term prognosis of NSSI patients depend to a large extent on good nurse-patient communication and support from the whole society, including the family and school environment. Effective nursing care, as well as patient compliance, has been shown to help improve the physical and mental status of NSSI patients.

**Figure 2:**
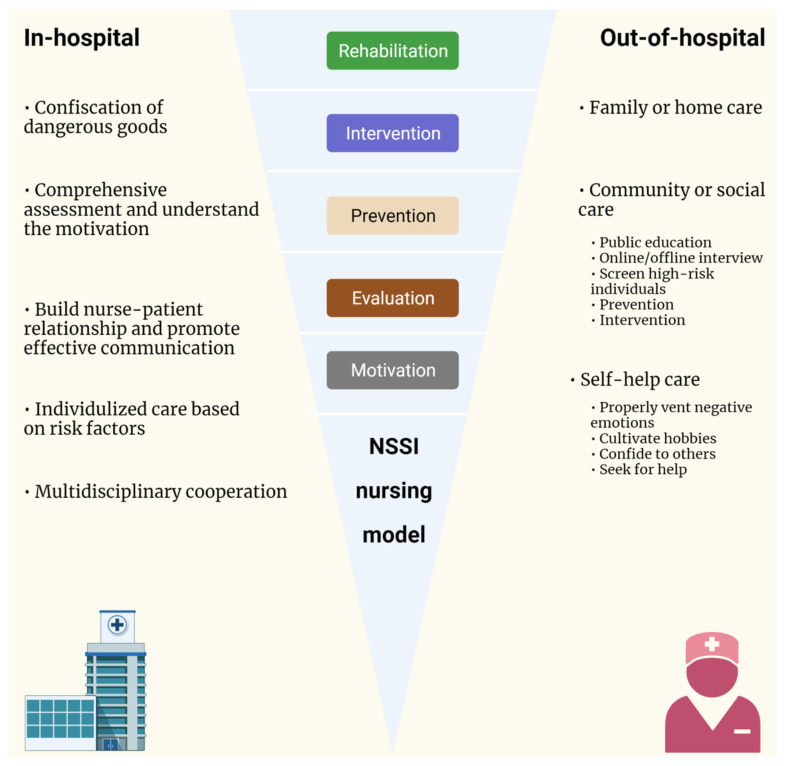
An integrative model of in-hospital and out-of-hospital nursing care for NSSI behavior. A step-wise, five-level model has been proposed, including motivation, evaluation, prevention, intervention, and rehabilitation. In-hospital and out-of-hospital nursing care for individuals with NSSI is a synergistic and coordinated continuum. For in-hospital conditions, immediate evaluation on the motivation and risk factors related to NSSI behaviors is the first step. Confiscation of dangerous goods helps to further reduce the risk of in-hospital NSSI events. Based on harmonious patient–nurse relationship, multidisciplinary collaboration can effectively deliver emergence interventions, handle psychiatric comorbidities, provide psychoeducation, and assess readiness for discharge. When returning home, living in the family and community environment needs more rehabilitative skills, peer support, and self-help, which, collectively, aims to reduce the rehospitalization rate and suicidal risk.

Based on previous NSSI-related study findings, it is recommended that when facing NSSI patients, the very first step for nursing care is to improve the communication skills of nurses and other caregivers, improve patients’ self-esteem, and change their improper cognitive behavior patterns by eliminating factors that may reinforce the NSSI behavior [5]. Meanwhile, mental health care services should integrate nursing roles, mental health care, and medical sociology into a systematic entity [68]. Following the PRISMA criteria, we performed a review on the nursing interventions on NSSI using the PubMed database. The search strategy was set with the following keywords: ((“Self-Injurious Behavior”[Mesh]) OR ((((((((((((((((((((((((((Behavior, Self-Injurious) OR (Self Injurious Behavior)) OR (Self-Injurious Behaviors)) OR (Intentional Self Injury)) OR (Intentional Self Injuries)) OR (Self Injury, Intentional)) OR (Intentional Self Harm)) OR (Self Harm, Intentional)) OR (Nonsuicidal Self Injury)) OR (Nonsuicidal Self Injuries)) OR (Self Injury, Nonsuicidal)) OR (Deliberate Self-Harm)) OR (Deliberate Self Harm)) OR (Self-Harm, Deliberate)) OR (Self-Injury)) OR (Self Injury)) OR (Non-Suicidal Self Injury)) OR (Non Suicidal Self Injury)) OR (Non-Suicidal Self Injuries)) OR (Self Injury, Non-Suicidal)) OR (Self Harm)) OR (Harm, Self)) OR (Self-Destructive Behavior)) OR (Behavior, Self-Destructive)) OR (Self Destructive Behavior)) OR (Self-Destructive Behaviors))) AND (((“Nursing”[Mesh])) OR (Nursings))) AND (((“randomized controlled trial”[pt] OR “controlled clinical trial”[pt] OR randomized[tiab] OR placebo[tiab] OR “drug therapy”[sh] OR randomly[tiab] OR trial[tiab] OR groups[tiab]) OR ((“clinical”[tiab] AND “trial”[tiab]) OR “clinical trials as topic”[mesh] OR “clinical trial”[pt] OR random*[tiab] OR “random allocation”[mesh] OR “therapeutic use”[sh])) OR (“cohort studies”[mesh] OR “case–control studies”[mesh] OR “comparative study”[pt] OR “risk factors”[mesh] OR “cohort”[tw] OR “compared”[tw] OR “groups”[tw] OR “case–control”[tw] OR “multivariate”[tw])). Although more than 2600 papers have been identified by January 31, 2023, no study has been performed exclusively to explore any specific nursing intervention on NSSI behaviors. Based on findings from epidemiological investigations and questionnaire surveys, we propose an integrative model of in-hospital and out-of-hospital nursing care for individuals with NSSI (Figure 2).

### 3.1. In-Hospital Care

#### 3.1.1. Understanding the Motivation for NSSI Behavior

The objective of in-hospital care is mainly for those with acute onset of NSSI behavior, but is not limited to adolescents. The definition of motivation is different from the aforementioned “risk factors”, and it mainly refers to mental activity that directly induces NSSI behavior, while risk factors seem to be the foundation or susceptibility of NSSI behavior. To implement effective nursing care, therefore, nurses need to understand the motivations of NSSI behavior with the applications of psychometric questionnaires.

Studies have shown that the most common motivation for individuals to exhibit NSSI behavior is to obtain relief from painful experiences such as grief, guilt, flashbacks, or depersonalization [4]. That is, the physical pain caused by the NSSI behavior itself can help the individual to ease bad feelings and regain a calm mind [69]. A survey showed that 103 of the 856 adolescent participants had a history of NSSI, and the most common reason for NSSI endorsed by these adolescents was to “get relief from a terrible state of mind” (agreement rate 79%) [70]. Some adolescents may think that they need to punish themselves through NSSI behavior, to receive more attention from their elders or classmates, or to make others feel guilty for themselves [4]. Another study showed that adolescents who saw the blood due to NSSI behaviors indulged in a sense of reality [71]. In addition, other motivations that can lead to NSSI behaviors include neuronal hyperexcitability and unhealthy interests [72].

Interestingly, a study published in 2020 explored the addictive nature of self-injurious behavior and found that both the rate of dopamine production and the concentration of dopamine were abnormally increased in the brains of patients with NSSI behaviors, which was similar to the mechanisms of addiction to psychoactive substances [73]. This phenomenon was also mentioned in an earlier study. Selby et al. speculated in 2012 that NSSI could lead to the release of endogenous opioid peptides in response to self-inflicted damage, thus resulting in the feeling of euphoria [74]. Therefore, the long-term and repeated NSSI behavior in some patients cannot be eliminated due to the addictive nature of the behavior itself.

#### 3.1.2. Standardized and Safe In-Hospital Management

Some scholars have already pointed out that systematic evaluation and treatment in a psychiatric hospital is recommended regardless of any preexisting psychiatric disorders that meet the relevant diagnostic criteria if patients report risk factors, such as personality characteristics, adverse family or social environment, having repeated occurrences of NSSI behavior within one year that led to moderate or severe damage to the body, suffering from severe depression, anxiety, other negative emotional experiences, and maladaptation to the environment [46]. Hospitalized patients should be strictly supervised by at least one clinically experienced nurse to avoid exposure to dangerous objects such as scissors, fruit knives, and glass that may be used for self-injury behavior. “Providing a safe hospital environment” by checking the belongings on admission, clearly informing the patient of the negative consequences of NSSI behavior, paying special attention to in-patient activities, and distracting the patient’s attention, was explicitly underlined in an Irish study using a semi-structured interview with psychiatric nurses [75]. When patients have NSSI thoughts or behaviors, notify the physician immediately and temporary restraining might be considered. For adolescent patients, nurses need to inform their guardians about the NSSI behavior; meanwhile, nurses also need to ensure whether the adolescents can obey their commitment to quitting NSSI behavior in the future [11].

Another study pointed out that nurses’ counter-transference in providing nursing services to NSSI patients can reduce nurses’ negative thoughts and behaviors, thereby improving nursing quality [76]. This suggests that nurses should first deal with their own negative emotions in a timely manner to optimize their thinking and behavior while providing mental health care for patients with NSSI.

In addition, given that NSSI potentially shares similar pathological manifestations to drug addiction, nurses should pay attention to the possible adverse consequences similar to drug addiction, such as serious infections, accidental death, and a higher risk of suicide, when caring for patients with NSSI behavior [73].

#### 3.1.3. Individualized Psychological Care

Individualized psychological care is also very important for NSSI patients. A study in China pointed out that nurses should guide NSSI patients to correct their false cognitive patterns and form a more objective and rational self-evaluation through individualized psychological care so that the patients can gradually accept themselves and achieve self-fulfillment [77]. First of all, nurses are required to be good listeners when patients with NSSI tend to express their pain or emotions [78]. In addition, nurses in the Department of Emergency or Psychiatry should assess the patients’ risk of more severe self-injurious behavior and accidental death and understand the differences in patients’ NSSI behavior in terms of presentation, characteristics, and function [79]. In other words, nurses can assist patients with NSSI behaviors in identifying the triggers for their self-injurious behaviors, and cooperate with them to develop optimized treatment strategies, with long-term monitoring of their self-injurious behaviors [79]. This nursing approach helps to improve the psychological status of patients with NSSI by helping to realize the defects in their thinking and behavioral patterns to shape a wholesome personality. Under this circumstance, the frequency of NSSI-related ideation and behavior will be gradually reduced and even eliminated.

Nurses should improve the humanity of nursing services to promote effective communication with patients. Patients who have NSSI behaviors or those admitted to the hospital due to NSSI behaviors are likely to need more medical resources and humanistic care. To establish a good nurse–patient relationship, nurses need to maintain patience when communicating with these patients [80,81]. Several studies have pointed out that the quality of nursing care received by patients after an NSSI event is closely related to their satisfaction with the hospitalization experience and their long-term mental well-being after discharge [82,83,84], i.e., nursing care is as important as psychiatric treatment for patients with NSSI behaviors. However, in some non-psychiatric departments such as the Emergency Department and Trauma Surgery that may also receive such patients, nurses often ignore or cannot adequately deal with the mental health problems of patients due to lack of time or psychological nursing experience [85], which can be manifested as decreased nurse–patient communication, low communication efficiency, or even lack of communication [86]. Several studies in recent years have assessed nurses’ attitudes towards “caring for patients with NSSI” and proposed several recommendations, such as improving nurses’ confidence and ability to provide care for patients with NSSI from the perspective of professional education and clinical supervision [87,88,89,90]. However, the number of studies in this area is still limited, and the effects of the proposed recommendations need to be further evaluated in clinical practice.

#### 3.1.4. Multi-Disciplinary Cooperation

It is noteworthy that a recent semi-structured interview with Danish nurses in an Emergency Department revealed that the lack of psychological nursing skills and a poor health care system are important reasons for unsatisfactory outcomes when caring for NSSI patients. Recently, an online survey carried out by Australian researchers also showed that nurses from both Emergency Department and Psychiatry Department were more accurate in their perception of NSSI, but nurses from Psychiatry Department were more confident in handling NSSI events because psychiatric nurses generally had more knowledge and clinical experience [91]. The way to solve this problem is not limited to improving the psychological care ability of nurses in the Emergency Department, but promoting close cooperation between the Emergency Department and Psychiatry Department through methods such as nursing consultation is recommended [92].

Other researchers point out that negative attitudes and experiences with nursing, lack of professional education and training, differences in perceptions of the role of healthcare personnel, diverse clinical cultures, and perceptions of NSSI behavior itself are the most relevant factors that influence the nursing efficacy of NSSI globally [93]. Therefore, to improve the nursing effect on NSSI patients from a long-term perspective, we should further promote multidisciplinary cooperation in the emergency and psychiatry departments and focus on nurse–patient interaction with patients with repetitive NSSI behaviors in the process of nursing training [91,94,95], aiming to build a comprehensive, systematic, scientific, humanized, and individualized physical and mental nursing care system within the hospital.

### 3.2. Out-Of-Hospital Care

#### 3.2.1. Home Care

Studies have shown that keeping in contact with parents or non-parent adults, as well as friends of the same age, can effectively reduce the incidence of NSSI behaviors in sexual minorities [47,96]. Indeed, building a cozy family environment is very important for NSSI patients, especially adolescents. A harmonious family atmosphere is conducive to promoting communication among family members, thereby helping adolescents to smoothly leave behind their psychological difficulties.

As mentioned previously, for patients in adolescence, their cognitive paradigm, thinking habits, behavioral patterns, and emotional regulation methods would be vulnerable to the subtle influence of their parents. Therefore, parents should set a good example in front of teenagers and choose appropriate ways to vent their negative emotions to avoid adverse effects on teenagers. Meanwhile, all family members are responsible for creating a harmonious family environment to avoid quarrels or fights, which helps to make a sense of security among teenagers [57].

#### 3.2.2. Social Care

Social care mainly refers to special education programs on mental health in schools and communities, as well as screening for certain factors that may contribute to NSSI behavior.

First of all, schools should strengthen the supervision and management of adolescents who may have NSSI behaviors and help to meet students’ psychological demands with a student-oriented attitude. Studies have found that a safe school environment is a strong protective factor for adolescents at high risk of NSSI [47]. Given that schools are the main place where teenagers learn to socialize, teachers should continuously pay attention to the emotional changes and psychological status of teenagers. If teenagers encounter setbacks or express negative emotions repetitively, teachers are responsible to provide special attention and immediate support and helping them to use the proper approaches to vent negative emotions, rather than hurting themselves [65]. When a student has aggressive behaviors against themselves or others, teachers should intervene immediately to prevent harmful behaviors, which may develop into NSSI if not appropriately solved.

Second, the community can invite nurses with nursing experience for patients with NSSI to impart knowledge on NSSI nursing through telephone interviews, home visits, or community public lectures. Meanwhile, relevant government departments should undertake responsibility for regularly and strictly reviewing popular books, newspapers, and online literary and artistic works that are accessible to adolescents, to prevent them from indiscriminately imitating NSSI-related content [65].

In addition, to identify individuals with high risk for NSSI behaviors as early as possible, schools can cooperate with local psychiatric hospitals or professional psychological evaluators to establish a real-time NSSI assessment system that can obtain and analyze data. The psychometric questionnaires adopted can be various, such as the “Ottawa Self-Injury Inventory”, “Modified Overt Aggression Scale”, “Life Event Scale”, “Questionnaire of Suicide Awareness”, “Beck Suicidal Ideation Scale”, and the “Self-rating Depression Scale”, which are widely used for subjective evaluation of mental health problems. Individuals who are classified as “moderate or high risk for NSSI behavior” should be paid special attention by teachers, family members, and community workers to prevent any NSSI behavior.

#### 3.2.3. Self-Care

Self-care of NSSI patients should also be emphasized. Even if filling out the scale accurately can help to screen individuals with high risks, the patients themselves are the most sensitive to mood swings and abnormal behaviors. If the patient still has full insight, the bad cognitive and behavioral pattern can be gradually corrected by changing the way of regulating negative emotions, and the frequency of NSSI behaviors will also decrease, consequently.

For adolescent patients, they can learn to understand the causes of negative emotions and effective ways to defuse uncomfortable feelings and increase their confidence in dealing with negative emotions correctly. When having difficulty dealing with negative emotions by themselves, adolescent patients should seek help in time, such as confiding in teachers, parents, or counselors [57]. The cultivation of personal interests and hobbies can also help adolescents to process bad emotions when necessary.

## 4. Perspectives

Adolescents will face many psychological difficulties, and healthcare professionals including nurses must be alert to the risk of developing mental disorders and NSSI behaviors. For nurses working in the Department of Emergency or Psychiatry, a thorough assessment of the patient’s NSSI behavior and history of mental illness (both personal and family history) is essential for individualized physical and psychological care. Nurses should comprehensively understand the motivation of patients admitted for or exhibiting NSSI behaviors and screen for risk factors that may lead to NSSI behaviors [13]. Two major concerns need to be addressed: one is to prevent the recurrence of NSSI behaviors by identifying and eliminating risk factors, and the other is to implement immediate interventions when NSSI behaviors occur.

Currently, the number of studies on the nursing care of patients with NSSI behaviors is still small. In addition, the size of research samples should be increased in future studies, and more rigorous methods should be adopted to exclude irrelevant factors that may affect the research results. The research object is suggested to be expanded, i.e., both patients and their family members should be involved in the research. Psychiatric nurses need to broaden the nursing strategies when caring for patients with NSSI to ensure that the patient is physically and mentally protected [75]. We also recommend that NSSI patients with suicidal risk should be differentiated from those without suicidal ideation so that more personalized care can be implemented.

With increasing attention paid to psychological problems, effectively caring for patients with NSSI behaviors has become one of the priorities of multidisciplinary treatment. NSSI behaviors are more common in adolescents whose cognitive patterns, emotions, and behavioral control have not yet fully developed, and they are more likely to be affected by internal or external factors that may induce self-injurious behaviors. If left untreated, it can lead to serious consequences, such as causing suicidal ideation or even suicidal behavior. A variety of factors, such as a previous history of mental illness, family education, and social support, can contribute to the occurrence and development of NSSI behaviors. Timely and systematic nursing care can help to improve the prognosis of patients. Nursing care for NSSI patients is not only limited to in-hospital conditions but can also be extended to out-of-hospital circumstances. Nurses are mainly responsible for in-hospital nursing care, but due to a lack of basic knowledge and clinical experience in psychological support, the current situation of nursing for inpatients with NSSI behaviors needs more improvement. To further address the issue, an endeavor is needed to build a complete and targeted physical and mental nursing care system for NSSI from a multidisciplinary cooperation basis. The subjects of out-of-hospital care include the patient’s family, school, community, and the patient, all of whom need to work together to build an integrated NSSI nursing system covering prevention and post-event intervention. 

## 5. Conclusions

In conclusion, an integrative model of in-hospital and out-of-hospital nursing care for patients with NSSI behaviors helps to change the cognitive and behavior pattern and reduce the frequency of NSSI impulses, thereby facilitating long-term and comprehensive rehabilitation of social functions.

## Data Availability

No new data were created or analyzed in this study. Data sharing is not applicable to this article.
